# Clinical significance and role of coinfections with respiratory pathogens among individuals with confirmed severe acute respiratory syndrome coronavirus-2 infection

**DOI:** 10.3389/fpubh.2022.959319

**Published:** 2022-09-02

**Authors:** Ivelina Trifonova, Iva Christova, Iveta Madzharova, Svetla Angelova, Silvya Voleva, Ralitsa Yordanova, Tatiana Tcherveniakova, Stefka Krumova, Neli Korsun

**Affiliations:** ^1^National Laboratory “Influenza and ARD”, Department of Virology, National Centre of Infectious and Parasitic Diseases, Sofia, Bulgaria; ^2^Clinical Virology Laboratory, University Hospital “Prof. Dr. Stoyan Kirkovich”, Stara Zagora, Bulgaria; ^3^Clinic for Neuro Infections, Airborne, Roof, and Transmissible Infections, Infectious Hospital “Prof. Ivan Kirov”, Department of Infectious Diseases, Parasitology and Tropical Medicine, Medical University of Sofia, Sofia, Bulgaria

**Keywords:** coinfection, SARS-CoV-2, respiratory virus, viral load, clinical significance, COVID-19

## Abstract

**Introduction:**

This study aimed to determine the prevalence, viral profile, and clinical features of coinfections with severe acute respiratory syndrome coronavirus-2 (SARS-CoV-2) and other respiratory viruses.

**Methods:**

Nasopharyngeal samples and clinical data of 221 hospitalized patients and 21 outpatients were collected and analyzed. Real-time reverse transcription-polymerase chain reaction was used to detect SARS-CoV-2, influenza virus, respiratory syncytial virus (RSV), human metapneumovirus (HMPV), parainfluenza virus (PIV) 1,2,3, rhinovirus (RV), adenovirus (AdV), bocaviruses (BoV), and seasonal coronaviruses (OC43, 229E, NL63, and HKU1). Viral load was determined by capillary electrophoresis.

**Results:**

From November 2020 to mid-March 2022, 242 SARS-CoV-2 positive patients were tested for seasonal respiratory viruses, and 24 (9.9%) cases of coinfections were detected. The distribution of viruses involved in cases of coinfections were as follows: HMPV (*n* = 6; 25%), RSV (*n* = 4;16.7%), AdV (*n* = 4; 16.7%), BoV (*n* = 4; 16.7%), PIV3 (*n* = 2; 8.3%), influenza A (H3N2; *n* = 2; 8.3%), RV (*n* = 1; 4.62%), and RV+BoV (*n* = 1; 4.62%). The proportion of detected coinfections with SARS-CoV-2 was highest in children aged 0–5 years (59%), followed by those >65 years (33%). In specimens with detected coinfection, the viral load of influenza was higher than that of SARS-CoV-2, and the mean viral load of SARS-CoV-2 was higher than that of the other respiratory viruses. C-reactive protein (CRP) and lymphocytes count in co-infected patients >65 years of age were on average higher than in children <16 years of age (mean CRP of 161.8 ± 133.1 mg/L; 19.7 ± 3.09% vs. mean 6.9 ± 8.9 mg/L, 0.9 ± 3.1%; *p* < 0.01). Patients >65 years of age co-infected with SARS-CoV-2 and other respiratory viruses had longer hospital stays than those <16 years of age (mean 9 ± 3.96 days vs. 5.44 ± 1.89 days; *p* = 0.025). The combination of AdV and SARS-CoV-2 is fatal for patients aged >65 years.

**Conclusion:**

In patients aged >65 years, coinfection with SARS CoV-2 and other respiratory viruses, together with concomitant diseases, causes worsening of the clinical picture and complications, and can be fatal. Screening of patients with SARS CoV-2 for other respiratory viruses is needed to select appropriate treatments and prevent a fatal outcome of the disease.

## Introduction

Acute respiratory infections (ARIs) cause significant morbidity and are associated with a large number of medical consultations, hospitalizations, and high medical and social costs. Over 200 species of viruses from different families with similar transmission mechanisms, tissue tropism, and clinical manifestations have been identified as etiological agents of acute upper and lower respiratory tract infections. Some respiratory viruses originating from the animal kingdom may acquire the ability to cause diseases in humans and spread rapidly in human society due to the lack of immunity in the human population, causing pandemics (1). Severe acute respiratory syndrome coronavirus-2 (SARS-CoV-2), which emerged at the end of December 2019 in China, has become widespread globally, causing the largest pandemic in the last 100 years, with an enormous number of human casualties and serious consequences in many areas of human society (2).

Bulgaria has been hit hard by the coronavirus disease (COVID-19) pandemic, which caused more than 1.1 million infections and 36 950 related deaths as of April 2022 Given that Bulgaria's population is 6.5 million, we can conclude that the pandemic has left its mark on the country's demographics and is a challenge to the health system.

Strict non-pharmaceutical preventive interventions (social distancing, wearing face masks, reduced travel, closing schools and businesses, and hand hygiene) implemented to mitigate the diffusion of SARS-CoV-2 have had a significant impact on the prevalence and some aspects of the epidemiology of other respiratory viruses, such as the virus, age, and monthly distribution (3). No influenza virus was isolated from the country during the 2020–2021 season. In the following 2021–2022 season, the circulation of influenza viruses was significantly lower than in the pre-pandemic seasons, with a later peak incidence, which coincided with a period of loosening of the public health measures in March 2022.

The emergence of the new coronavirus against the background of an unusual decrease in the prevalence of respiratory viruses poses a new challenge to the laboratory team, as to whether co-infections between the new coronavirus and other respiratory viruses are possible, how often they occur, and how they affect the severity of the disease. In the first year of the pandemic, research in many laboratories in the country focused only on detecting the pandemic coronavirus, which precluded the diagnosis of other respiratory viral pathogens causing infections with similar clinical manifestation. Information on the prevalence of seasonal respiratory viruses in Bulgaria is very limited, despite published data on possible coinfections between SARS CoV-2 and other respiratory viruses (4). SARS CoV-2 causes a wide range of clinical manifestations ranging from asymptomatic or mild symptoms to severe or even critical illness and death (5). According to a number of researchers, coinfection between SARS CoV-2 and other respiratory viruses can induce a more severe inflammatory process, which can aggravate the state of illness and cause unfavorable outcomes (6). Coinfections can be associated with an increased rate of complications, longer treatment duration, a higher rate of intensive care unit (ICU) admission, and mortality compared to a single infection (7, 8). However, other authors believe that coinfections with additional respiratory viruses do not worsen clinical severity and outcome. This is probably due to viral interference when one virus inhibits the replication of another virus through resource competition, by induction of interferon, or other immunological mechanisms (9). Early detection of viral coinfections may be helpful for patient management and prognosis and can prevent unnecessary use of antibiotics. The present study aimed to explore the co-infections with SARS-CoV-2 and other respiratory viruses: to identify the respiratory viruses involved, to determine the possible link between the age of patients and the frequency of co-infections as well as the differences in viral loads of detected viruses in cases of coinfections and clinical manifestation in co-infected and monoinfected patients. The possible link between the occurrence of coinfections and the deterioration of the clinical picture in patients from different age groups is another aim of the study. Despite numerous publications on the subject, the question of the clinical relationship between co-infections remains unclear (10–14).

## Materials and methods

### Study population and specimen sampling

Nasopharyngeal swabs from patients with confirmed SARS-CoV-2 infection, together with clinical and epidemiological data, were prospectively collected from both inpatients and outpatients. Hospitalized patients were treated in two hospitals located in distinct geographic regions. The swabs were placed in 2 ml medium (polyester collection swabs), stored on ice for transport to the national testing laboratory, and the swabs were stored at 4°C for up to 72 h before being sent to the laboratory. The samples were processed immediately or stored at −80°C before testing.

### Nucleic acid extraction and reverse transcription polymerase chain reaction (RT-PCR)

Viral DNA and RNA were extracted by an automatic extraction system using the ExiPrep Dx Viral DNA/RNA kit (Bioneer, Daejeon, Republic of Korea) in accordance with the manufacturer's instructions. SARS-CoV-2 was detected by real-time RT-PCR using the TaqPath COVID-19 CE-IVD PCR Kit (Thermo Fisher Scientific). This kit specifically identifies three regions in the SARS-CoV-2 genome: N-gene, S-gene, and ORF ab. It has a clinical sensitivity of 100% [95% confidence interval (CI): 97.9–100.0%] and clinical specificity of 100% (95% CI: 98.6–100.0%). The detection limit was 250 copies/ml. Amplification was performed using the QuantStudio™ 5 real-time PCR system, 96-well (ThermoFisher Scientific).

SARS CoV-2-positive specimens were tested for influenza type A and B viruses, as well as for eight other seasonal respiratory viruses: respiratory syncytial virus (RSV), human metapneumovirus (HMPV), parainfluenza virus (PIV) types 1/2/3, rhinovirus (RV), adenovirus (AdV), bocavirus (BoV), and four seasonal human coronaviruses: OC43, 229E, NL63, and HKU1. Influenza A viruses are differentiated into A (H1N1) pdm09 and A (H3N2). Screening for influenza and non-influenza respiratory viruses was performed using singleplex real-time RT-PCR assays with the SuperScript III Platinum^®^ One-Step qRT-PCR system (Invitrogen, Thermo Fisher Scientific, Waltham, MA, USA). The primers and probes used in this study were identical to those previously described (15–17). Amplification was performed using a CFX96 thermal cycler (Bio-Rad Laboratories, Inc., Hercules, CA, USA). A cycle threshold (Ct) value <38 was considered positive. The thermocycling conditions for real-time PCR assays are listed in Table 1.

**Table 1:** Thermocycling conditions of real time polymerase chain reaction for detection of severe acute respiratory syndrome coronavirus-2, eight common respiratory viruses, four seasonal human coronaviruses, and influenza viruses.

***Thermocycling conditions of Real Time RT-PCR for detection of SARS-CoV-2***		
Reverse transcription	53°C	10 min
Initial denaturation	95°C	2 min
Amplification - 40 cycles	95°C	3 sec
	60°C	30 sec
***Thermocycling conditions of Real Time RT-PCR for detection of RSV, HMPV, PIV 1,2,3, RV, AdV and BoV***		
Reverse transcription	45°C	10 min
Initial denaturation	95°C	10 min
Amplification - 44 cycles	94°C	30 sec
	57°C	1 min
***Thermocycling conditions of Real Time RT-PCR for detection of coronaviruses OC43, 229E, NL63 and HKU1***		
Reverse transcription	48°C	20 min
Initial denaturation	95°C	5 min
Amplification - 44 cycles	95°C	15 sec
	55°C	1 min
***Thermocycling conditions of Real Time RT-PCR for detection of influenza viruses***		
Reverse transcription	50°C	30 min
Initial denaturation	95°C	2 min
Amplification - 44 cycles	94°C	15 sec
	56°C	30 sec

### Quantitative methods

Quantitative capillary electrophoresis was used to determine the viral load of each viral pathogen involved in the coinfection with SARS-CoV-2. The method was implemented using a QIAxcel Advanced capillary system. Gel cartridges were used in combination with a preprogrammed method, allowing the separation and analysis of DNA. The preparatory stage of the method included the amplification of genomic targets of the individual respiratory viruses using a commercial kit (Seeplex^®^ RV15 OneStep ACE Detection) containing the necessary primers. The SuperScript III Platinum One-Step qRT-PCR System (Invitrogen, Thermo Fisher Scientific, Waltham, MA, USA) and suitable primers were used to amplify the RdRp region of the SARS-CoV-2 genome.

A formula converting the concentration of viral amplicons into viral copies was used:


$$\begin{array}{l} Number\;of\;copies\;of\;DNA\;template\;of\;ul=\;\frac{\left(DNA\;concentration\;(ng/\mu l)\times \;Avogadro^{{}^{\prime }}s\;number\right)}{\left(template\;length\;(bp)\times \;conversion\;factor\;to\;ng\;\times \;average\;weight\;of\;base\;pair\;(Da)\right)}\\ \;Number\;of\;copies=\;\frac{\left(DNA\;concentration\;(ng/\mu l)\times \;(6.022\;\times \;10^{23})\right)}{(pattern\;length\;(bp)\;\times \;[1\times 10^{9}]\times \;660)\;} \end{array}$$

To determine the concentration of the amplicons (ng/μl) and the length of the amplified region of the viral genome [base pairs (bp)], we used capillary electrophoresis.

We had in mind that the mass of the DNA nucleotide was 330 Da, and the mass of the bp in the double-stranded DNA was 660 Da. In this formula, the weight of the DNA nucleotide was 330 g, and the weight of the bp formula in double-stranded DNA was 660 g. In this expression, the number of moles present in 1 g of material is inversely proportional to the weight. Avogadro's number, 6.022 × 10^23^ molecules/mol, was used to calculate the number of molecules per gram. The number of copies of the template in the sample was obtained by multiplying by 1 × 10^9^ to convert to ng/μl and then multiplying by the amount of template (in ng/μl).

### Clinical data and definitions

The assessment of patients was based on the assessment of the condition of patients using a category scale (8, 18).

1- is not hospitalized with resumption of normal activity;
2- is not hospitalized, but cannot resume normal activities;
3- hospitalized, not requiring additional oxygen;
4- hospitalized, requiring additional oxygen;
5- hospitalized requiring high-flow nasal oxygen therapy, non-invasive mechanical ventilation, or both;
6- hospitalized, requiring ECMO, invasive mechanical ventilation, or both;
7- death.

We used data from the described epicrisis for each doctor, with a specialist in the field participating in the analysis. The patient's medical record presented demographic data, comorbidities presenting symptoms and signs, blood biochemical indices, use of antivirals and antibiotics, corticosteroids and bronchodilator drugs, intensive care, length of hospital stay, need for artificial ventilation, and adverse events classified according to the criteria of the National Cancer Institute for adverse events, version 4.0.

Pneumonia was determined by X-ray examination and the presence of pulmonary infiltrates. Acute respiratory distress is defined again by the definition of Berlin (19).

### Statistical analysis

Chi-square or Fisher's exact tests were used to assess categorical variables, which were presented as total counts and percentages. Continuous variables were compared using a paired comparison plot, Fisher LSD, *t*-test, and Mann–Whitney *U*-test. Differences with *p*-values < 0.05 were considered as statistically significant. The statistical software package Origin: Data Analysis and Graphing Software was used. The Cox regression method is also applied, according to the *p*-value it is determined accordingly whether the regression coefficient is significantly different from zero.

## Results

### Characterization of the group of studied patients

This study covered the period from November 2020 to mid-March 2022 and included 242 SARS-CoV-2 positive patients, of which 221 (91.32%) were hospitalized and 21 (8.7%) were outpatients. Bases for sampling were two hospitals - Infectious Diseases Hospital “Prof. Ivan Kirov” in Sofia where 148 of study patients were treated and University Hospital “Prof. Dr. Stoyan Kirkovich”, Stara Zagora with 73 study patients. Fifteen (6.2%) patients were admitted to the ICU. The mortality rate of hospitalized patients was 9.5%. Outpatient specimens were collected from the hospital emergency center. Patient age varied from 10 days to 90 years (mean age 47 ± 32.11 years; median age 65 years). The distribution of patients by age was as follows: 55 (22.7%) were 0–5 years old, 27 (11.2%) were 6–16 years old, 36 (14.9%) were 17–64 old, and 124 (51.2%) were >65 years old. The number of male patients studied (121) was equal to that of female patients.

### Identification of coinfections in SARS-CoV-2 positive patients

Among the 242 patients, who were diagnosed with COVID-19, coinfection with other respiratory viruses was detected in 24 (9.9%) patients: 23 (95.8%) were coinfected with a single respiratory virus, and one (4.2%) was coinfected with two viruses. The proportions of coinfections among SARS CoV-2 positive inpatients and outpatients were 21/221(9.5%) and 3/21 (12.5%), respectively. Among the coinfected patients, one (4.2%) required ICU admission and another (4.2%) had a fatal outcome. HMPV was the most commonly identified co-detecting virus (*n* = 6, 25%), followed by RSV, AdV, and BoV, which were identified with equal frequency (*n* = 4, 16.7%). The combinations of SARS CoV-2 with other pathogens, PIV3 (*n* = 2, 8.3%), influenza A (H3N2; *n* = 2, 8.3%), and RV (*n* = 1, 4.62%), were found relatively less frequent. SARS-CoV, RV, and BoV were detected only in triple infections.

To evaluate the co-circulation of the four seasonal human coronaviruses during the COVID-19 pandemic, we examined 155 (64%) of the patients. In this target group, co-infections between SARS-CoV-2 and the OC43, NL63, 229E, and HKU-1 viruses were not found.

### Seasonal and age distribution of detected coinfections in SARS-CoV-2 positive patients

**Figure 1:**
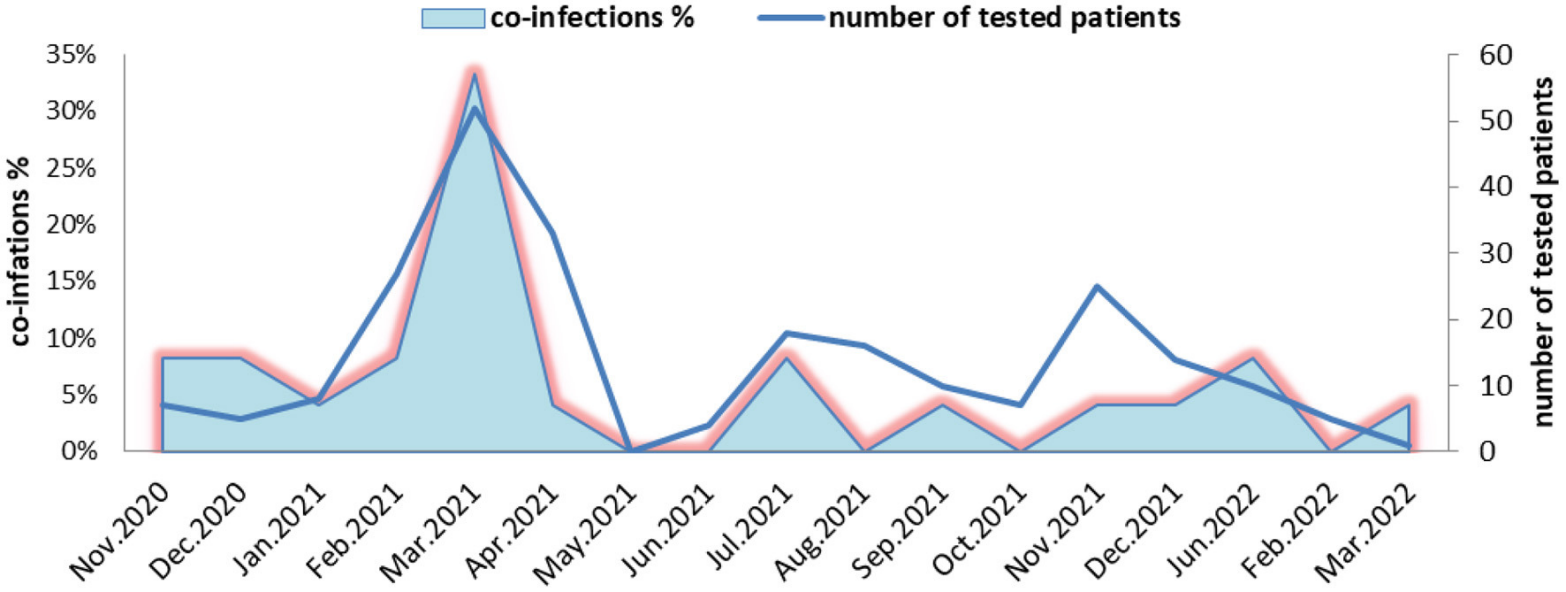
Monthly distribution of detected coinfections of severe acute respiratory syndrome coronavirus-2 and other respiratory viruses in Bulgaria during the period of November 2020 to March 2022.

During the COVID-19 pandemic, the highest number of coinfections with SARS-CoV-2 and other viral respiratory pathogens was identified in March 2021 (51/242, 33%), followed by those detected in November 2020, February 2021, July 2021, and January 2022, with detection rates of 8.3%. In December 2020, combinations of SARS-CoV-2 with AdV and SARS-CoV-2 with RSV were more frequently identified, and in September 2021 and December 2021, coinfections between SARS-CoV-2 and HMPV were more frequent. No influenza virus was detected in Bulgaria during the 2020/2021 season. In the 2021/2022 season, only influenza A (H3N2) virus circulated in the country. Two cases of coinfections between SARS-CoV-2 and influenza A (H3N2) were detected in January and March 2022 (Figure 1).

**Figure 2:**
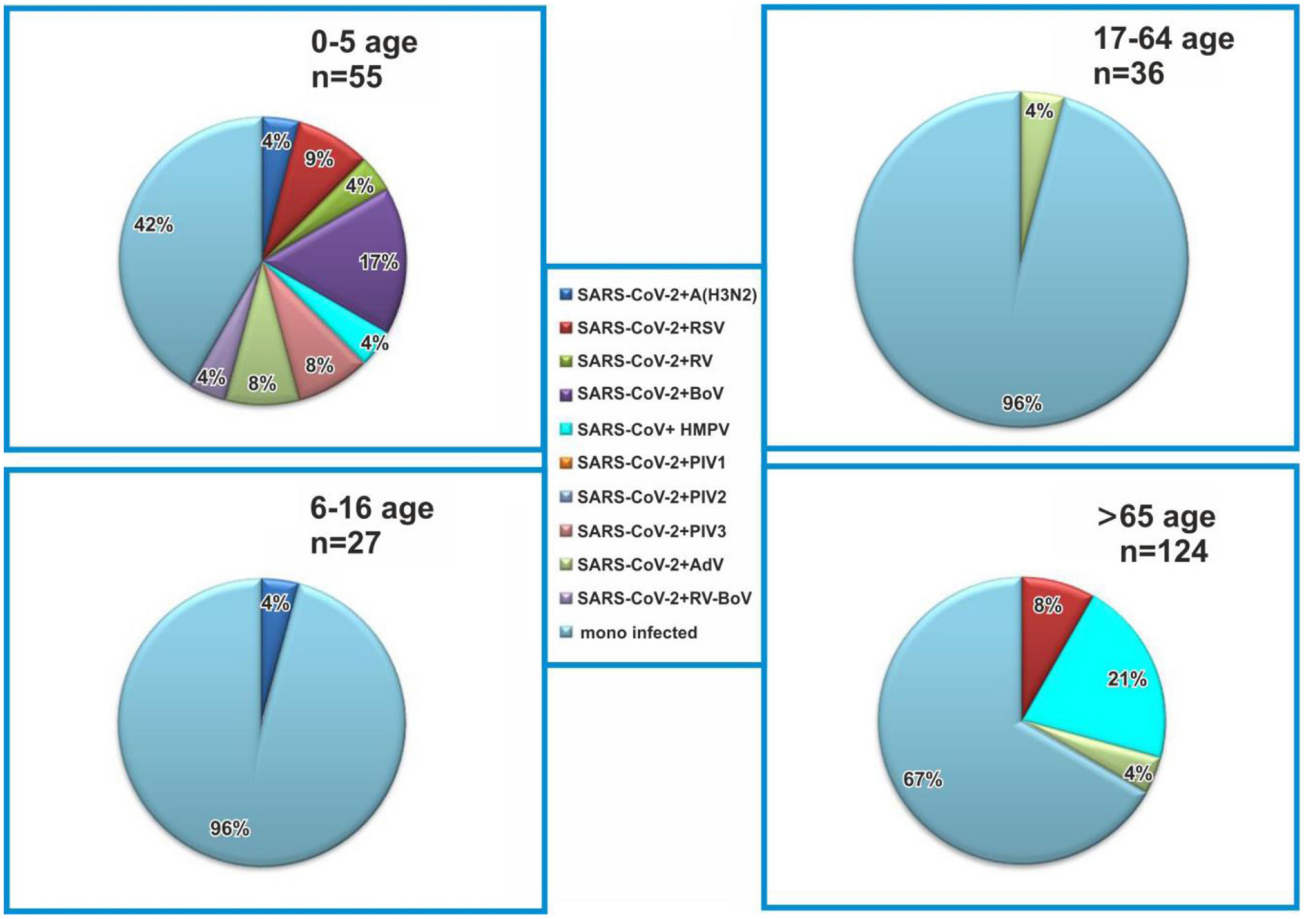
Age distribution of coinfected patients with severe acute respiratory syndrome coronavirus-2 (SARS-CoV-2) and other respiratory viruses. Monoinfected patients are positive for SARS-CoV-2.

In this study, coinfections were detected in SARS CoV-2-positive patients from all age groups (0–5, 6–16, 17–64, and 65+ years), and proportions of coinfections were 59, 4, 4, and 33%, respectively (Figure 2). The rate of coinfections was highest in the youngest patients (0–5 years), accounting for 25.4% of all cases in this age group, followed by adults >65 years (6.5%). Coinfections with SARS-CoV-2 and other respiratory viruses were less common in patients aged 5–16 years (3.9%) and 17–64 years (2.8%). The combination of SARS-CoV-2 and BoV was detected in the youngest children (16.7%), whereas in other age groups, no cases of this coinfection were detected. In the oldest patients, coinfections with HMPV were the most frequent (20.8%), while in children aged 0–5 years, they accounted for 4.7%. Two 13- and 3-year old patients were diagnosed with coinfections, including influenza A (H3N2) virus, with a detection rate of 4.7% for all coinfections, including SARS-CoV-2.

### Viral load of respiratory viruses involved in coinfections with SARS-CoV-2 determined by quantitative capillary electrophoresis

**Figure 3:**
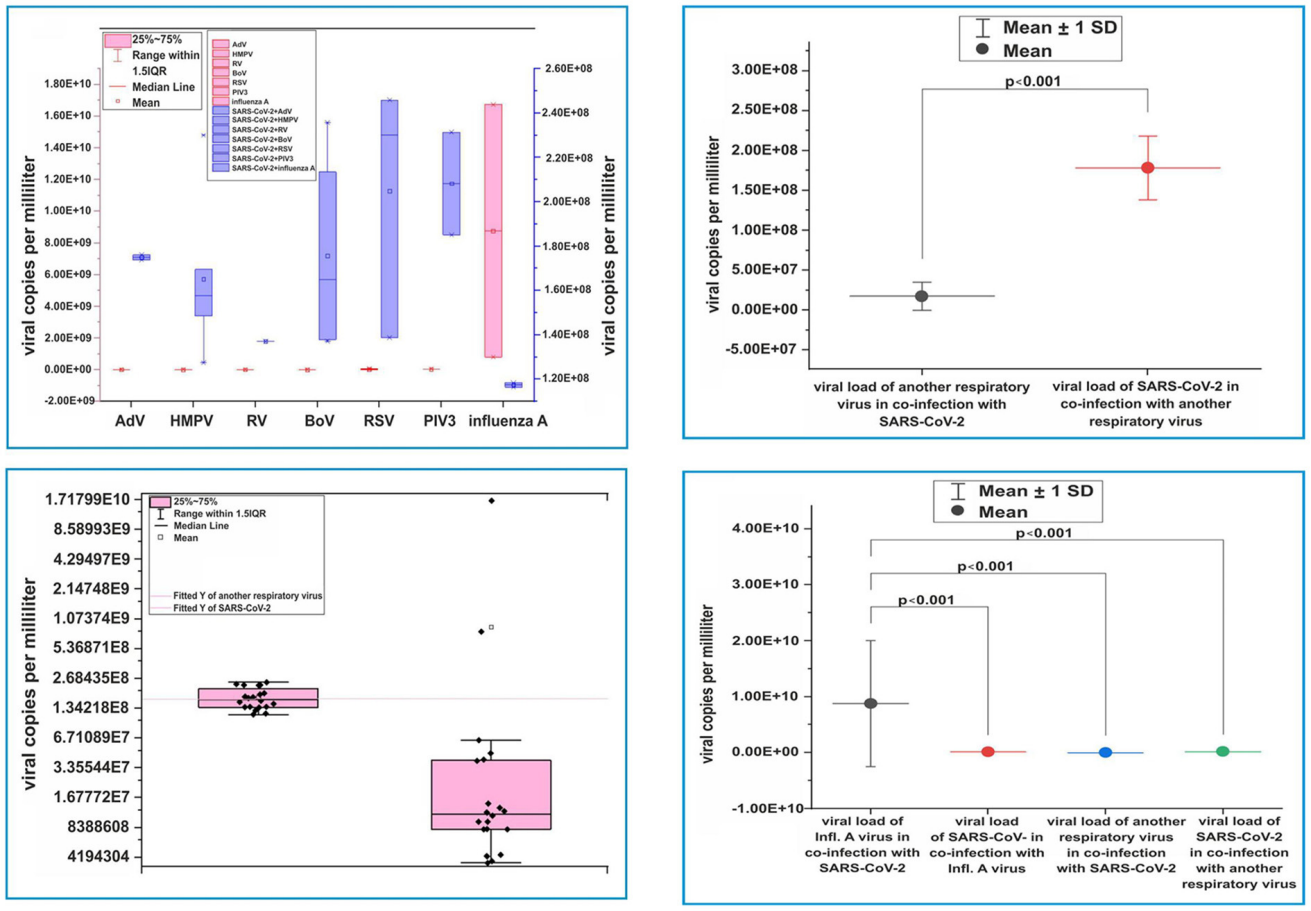
Virus load distribution. (**A**) Virus load distribution of severe acute respiratory syndrome coronavirus-2 (SARS-CoV-2) and adenovirus, human metapneumovirus, rhinovirus, bocaviruses, respiratory syncytial virus, parainfluenza virus, and influenza A (H3N2) virus, respectively, in cases of coinfections. (**B**) Distribution of viral load from SARS-CoV-2 and other respiratory viruses in monoinfections with SARS-CoV-2. (**C**) Comparison of viral loads of SARS-CoV-2 and other co-detecting respiratory viruses. (**D**) Comparing the viral load of influenza A virus with that of SARS-CoV-2 when discovered together in coinfections, SARS-CoV-2 when it is coinfected with other non-influenza viruses, and SARS-CoV-2 in monoinfections. Viral load was determined using capillary electrophoresis and a formula to convert the concentration to viral copies per milliliter. Virus copy values per milliliter are represented by dots. Mean values of viral copies per milliliter, median, and range 25–75%, distribution of viral copies are shown. Values were calculated using the Mann–Whitney U-test.

In this study, we compared the viral loads of SARS-CoV-2 with those of other respiratory viruses (HMPV, RSV, RV, AdV, BoV, and PIV3) involved in coinfections. We found that the mean value of the SARS-CoV-2 viral load (1.79E8 ± 4.00 virus copies/ml; Figure 3B) was higher than that of the six co-detecting respiratory viruses (1.74E7 ± 1.77 virus copies/ml; *p* < 0.001; Figure 3C). In patients with influenza A (H3N2)/SARS CoV-2 coinfection, the mean influenza viral load (8.77E9 ± 1.12E10 viral copies/ml) was higher than that of SARS-CoV-2 (1.17E8 ± 1.41E6 viral copies/ml; *p* < 0.001; Figure 3D). We compared the viral loads of the six detected respiratory viruses in patients infected with SARS-CoV-2 with those of influenza viruses and found significant differences (*p* < 0.001). Accordingly, influenza viruses had a higher viral load than those of the six respiratory viruses involved in coinfections with SARS-CoV-2 (1.75E7 ± 1.77E7 viral copies/ml vs. 1.17E8 ± 1.41E6 viral copies/ml; Figure 3A).

### Clinical data of study patients

Demographic, clinical, treatment, and laboratory data of the study patients were extracted from medical records. In children and adolescents <16 years of age, no sharp deterioration in the clinical picture or progression to a severe form of COVID-19 was observed. In co-infected children with SARS-CoV-2 and other respiratory viruses, the saturation was normal, and frequently observed symptoms were fever in the range of 37.4°-40°C (89%) and diarrhea (11%). In patients >65 years of age, the clinical manifestations of COVID-19 were more pronounced, with the most common being dyspnea, cough, fatigue, loss of appetite, and some with diarrhea. In this age group, the proportion of patients with concomitant diseases was 70%, and the most common were hypertension (*n* = 24, 48.9%) and diabetes (*n* = 12, 24.5%). In hospitalized patients >65 years of age, the incidence of cases with dyspnea was 31.4%, dyspnea with cough was 58.3%, and a combination of two symptoms and intense treatment was 57.1%. A total of 15 (21.4%) patients >65 years of age needed intensive treatment, and four (26%) of them had concomitant hypertension. All hospitalized patients who received oxygen therapy (*n* = 38, 17.2%) were >65 years old. Over 84% of bedridden patients underwent antibiotic treatment. Out of 221 hospitalized patients with confirmed SARS-CoV-2 infection, fatal outcomes were reported in 24 (10.4%), and coinfection with AdV was detected in one (4.3%) of them. Co-infected patients >65 years of age with SARS-CoV-2 and other respiratory viruses required longer hospital stays (mean 9 ± 4.33 days) compared to those <16 years of age (5.44 ± 2.01 days; *p* = 0.05). From the Cox regression results presented in Table 2, it is clear that there is an 11.9% increase in the risk of death with increasing time from disease onset to hospitalization (or an estimated risk of 0.83 times higher per day in patients with COVID-19 who did not seek timely hospitalization, *p* = 0.004). The probability of fatal outcome was increased in co-infected patients in contrast to mono-infected patients by 25.3% (this result did not have statistical significance *p* = 0.37). The 41.3% increased probability of death in intensive care patients was statistically significant (*p* = 0.002).

**Table 2:** Cox regression modeling of survival of patients with COVID-19 vs. time from disease onset to hospitalization, in co-infected patients and those requiring intensive care.

**Factor**	**Coefficients**	**Lower 95% CI**	**Upper 95% CI**	**SE**	***z***	***p*-Value**	**Exp *(B)***	**Lower 95% CI**	**Upper 95% CI**
Priod from onset of illness to hospitalization	−0.18	−0.3	−0.06	0.06	2.89	0.004	0.83	0.74	0.94
Coinfected	−0.93	−2.97	1.12	1.04	0.89	0.374	0.4	0.05	3.06
Intensive treatment	1.42	0.54	2.3	0.45	3.17	0.002	4.13	1.72	9.95

### Comparison of patients with mono- and coinfections

**Figure 4:**
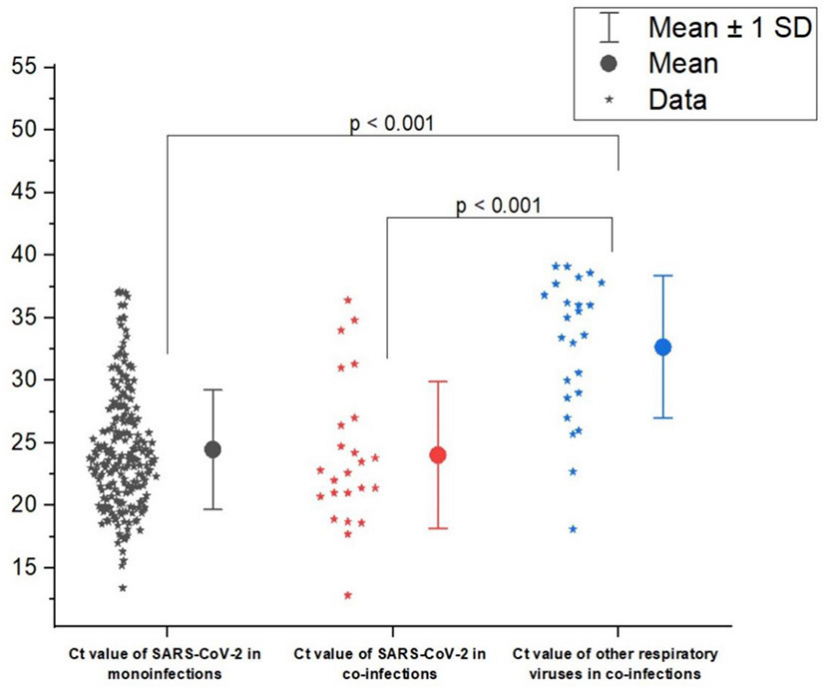
Distribution of cycle threshold (Ct) values of severe acute respiratory syndrome coronavirus-2 (SARS CoV-2) in cases of mono- and coinfection of SARS-CoV-2 and other respiratory virus. Ct was determined for the gene *ORFab* of SARS-CoV-2. Ct values are represented by asterisks. Mean Ct values, median, and range 25–75% are shown, see Ct distribution. Values were calculated with the Mann–Whitney *U*-test.

The Ct value provides a reliable means to quantify pathogen activity (a low Ct value corresponds to a high viral load in nasopharyngeal swabs). We compared the Ct values for SARS CoV-2 detection in patients with SARS CoV-2 mono- and coinfections. The mean Ct values did not differ significantly in cases of mono- and coinfections (24.46 ± 4.77 and 24.02 ± 5.84, respectively). However, the mean Ct value of SARS-CoV-2 was higher than that of the other respiratory pathogens in cases of coinfections (32.62 ± 5.69 vs 24.02 ± 5.84; *p* < 0.001; Figure 4).

The age of patients with mono- and coinfections, as well as some clinical and laboratory parameters, were also compared. The average age of patients with detected coinfections (26.9 ± 32.9 years) was younger in comparison to that of patients who only had SARS-CoV-2 infection (48.9 ± 31.5 years), and this difference was statistically significant (*p* < 0.001).

In terms of clinical symptoms, fever, fatigue, and diarrhea (Table 3) were more frequently observed in patients with coinfections compared to those with only SARS-CoV-2 infections (*p* < 0.05). In coinfected febrile patients, the mean body temperature was higher than that in the infected patients (38.7° ± 0.7°C vs 37.9° ± 0.6°C; *p* < 0.001). We analyzed the relationship between fever and the age of patients with mono- and coinfections. The patients >65 years of age who were co-infected had a mean body temperature of 38.9° ± 0.6°C, while the monoinfected patients in this age group had average body temperature values of 37.9° ± 0.1°C (*p* < 0.01). In children, there were no significant differences in body temperature between the coinfected and monoinfected children.

**Table 3:** Clinical manifestations, treatment, and clinical adverse events in patients with severe acute respiratory syndrome coronavirus-2 mono- and coinfection with other respiratory viruses.

	**SARS CoV-2 monoinfection (*n* = 218)** **(*n*, %)**	**SARS CoV-2 coinfection with other respiratory virus (*n* = 24) (*n*, %)**	***p*-Value**
**Symptoms**			
Fever	39 (17.9)	14 (58.3)	<0.0001
Fatique	24 (11)	8 (33.3)	0.0022
Shortness of breath	22 (10.1)	4 (16.7)	0.3235
Wheezing	25 (11.5)	3 (12.5)	0.8807
Cough	29 (13.3)	6 (25)	0.1220
Diarrhea	5 (2.3)	3 (12.5)	0.0079
**Treatment**			
Corticosteroids	53 (24.3)	5 (20.8)	0.7048
Intravenous antibiotics	64 (29.4)	9 (37.5)	0.4095
Oxigen therapy	38 (17.4)	6 (25)	0.3615
Ventilation support	14 (6.4)	1 (4.2)	0.6636
**Complications and clinical outcomes**			
Acute hearth failure	10 (4.6)	0 (0)	0.2839
Admittance to ICU	14 (6.4)	1 (4.2)	0.6636
Fatal outcome	24 (11)	1 (4.2)	0.2959

Increased levels of CRP were observed in patients aged >65 years coinfected with both SARS-CoV-2 and other respiratory virus (mean value 161.8 ± 133.1 mg/L). In patients aged <16 years, the average CRP level was 6.9 ± 8.9 mg/L (*p* < 0.01). The differences in CRP levels between the two age groups in patients with monoinfection were also statistically significant (*p* = 0.042). Accordingly, higher mean CRP levels were reported in monoinfected patients >65 years (123.4 ± 94.7 mg/L) compared to those in children <16 years of age (35.2 ± 71.6 mg/L). The differences in CRP levels in the blood of mono- and coinfected patients >65 years of age were not statistically significant (mean 123.4 ± 94.7 and 161.8 ± 133 1 mg/L, respectively). In hospitalized children and adolescents <16 years of age with detected coinfection between SARS-CoV-2 and other respiratory viruses, the mean percentage of lymphocytes was 19.7 ± 3.09%, while in those >65 years of age, lymphocytes count were 10.9 ± 3.1%, with the norm of 20–40% (*p* < 0.01). Comparison of the laboratory clinical data in mono- and coinfected patients is presented in Table 4.

**Table 4:** Laboratory data in coinfected patients with severe acute respiratory syndrome coronavirus-2 and respiratory viruses aged <16 and >65 years.

	** <16 years age SARS-CoV-2 co-infection with other respiratory viruses (*n* = 15)**	**>65 years age SARS-CoV-2 co-infection with other respiratory viruses (*n* = 8)**	***p*-Value**
Length of stay in hospital (days, mean)	9	5,44	0.05
PO_2_ (mmHg, mean)	96	85,2	0.018
Lym (%, mean)	19,7	10,9	<0.01
CRP (mg/L, mean)	161,8	6,9	<0.01

## Discussion

Coinfections with SARS CoV-2 and bacterial, fungal, or viral pathogens may aggravate clinical conditions and pose challenges to the diagnosis, treatment, and prognosis of the disease. In this study, we investigated the prevalence of coinfections of SARS CoV-2 with seasonal respiratory viruses, the profile of co-detecting pathogens, and the epidemiological, laboratory, and clinical characteristics of these infections. We tested 242 SARS CoV-2 positive patients for the presence of 15 types of seasonal respiratory viruses and identified 24 (9.9%) cases of coinfections. Previous studies in Bulgaria from the prepandemic period reported a 10.2% rate of coinfections among all-age population studied (20). Multiple authors in other countries have reported the presence of coinfections during the ongoing COVID-19 pandemic (21, 22). The proportion of coinfections in SARS-CoV-2-infected patients and the types of viruses involved vary in different regions of the world depending on the sensitivity of the diagnostic tests used, population studied, climate, sampling period, and temporal variations in viral epidemiology. In a study conducted in March 2020 in California, coinfection with another respiratory pathogen was reported in 24 (20.7%) of 116 individuals with confirmed SARS-CoV-2 (23). However, in recent studies, the incidence of respiratory viral coinfections was lower because of the public health and social measures introduced to limit the spread of SARS-CoV-2 (24). Some authors hypothesize that competitive advantage may play a role in SARS-CoV-2 interaction with other respiratory viruses during coinfection, and this is one reason why the coinfection rate in SARS-CoV-2 patients is much lower (25). Sentinel surveillance, carried out also in California from May 10, 2020 to June 12, 2021, identified 23 (1.7%) coinfections among 1,373 individuals positive for SARS-CoV-2 (26). A systematic review and meta-analysis, including 140 studies, conducted from December 1, 2019 to March 31, 2021, reported an overall pooled proportion of SARS-CoV-2 coinfections with other respiratory viruses of 6.6% (95% CI: 5.5–7.6) (27).

In the present study, the youngest age group (0–5 years) showed the highest rate of coinfections with SARS-CoV-2, which is in line with other reports (28). A systematic review and meta-analysis reported significantly higher proportions of coinfection with influenza viruses among children (3.2%) than among adult patients (0.3%; *p* < 0.01) (29).

In this study, HMPV, RSV, AdV, BoV, PIV, influenza A (H3N2), and RV were found to be involved in coinfections with SARS CoV-2. The most commonly identified virus was HMPV, followed by RSV, AdV, and BoV. We identified AdVs as co-detecting pathogens in four cases of coinfection, including one case with a fatal outcome. No cases of coinfections with seasonal coronaviruses were found. Other studies have reported that HMPV and AdV are the most frequent viral agents in SARS-CoV-2-positive patients after RV infection (10, 28, 30, 31). A study in the UK showed that SARS-CoV-2 coinfections with AdVs were significantly associated with increased odds of death (32). In the above-mentioned study in California, rhinovirus/enterovirus (6.9%), respiratory syncytial virus (5.2%), and non–SARS-CoV-2 Coronaviridae (4.3%) have been most frequently identified (23). Other authors have also reported the highest incidence of mixed infections with RV/EV in SARS CoV-2 infected patients (10, 22, 26, 33). In the present study, only one coinfection including RV, was detected. The incidence of SARS CoV-2 coinfections with influenza viruses was also very low. During the COVID-19 pandemic in Bulgaria, the circulation of influenza viruses was unusually weak compared to the prepandemic seasons, and only two (0.8%) cases of coinfections with SARS CoV-2 were identified in 2022. Similar observations have also been reported in other countries. A systematic review and meta-analysis of 11 prevalence studies conducted during the first wave of the COVID-19 pandemic reported 0.8% prevalence of influenza viruses in patients with confirmed COVID-19 (34). In a large pan-India study, including 13,467 samples tested from July 4, 2021 to January 31, 2022, only five (0.04%) cases of SARS-CoV-2/influenza virus coinfections were detected (35). The general opinion is that public health efforts to control the spread of SARS CoV-2 led to a reduction in influenza virus transmission (31). Because the pathogenesis of both viral infections and receptor use differ, there is no receptor competition or viral interference, suggesting increased airway inflammation and aggravation of the clinical picture (36, 37). An animal model of SARS CoV-2/influenza coinfection demonstrated increased disease severity among hamsters coinfected with SARS-CoV-2 and influenza A compared to those with SARS-CoV-2 monoinfection (37). In our study, one case of influenza virus/SARS CoV-2 coinfection was a hospitalized patient and the other was an outpatient. The high viral load of influenza viruses in nasopharyngeal swabs confirms active infection and high infectivity. Fast and accurate diagnosis of influenza infection is important because of the increased risk of disease exacerbation and the need for early treatment with antiviral drugs.

To investigate the intensity of viral replication during coinfection, we compared the viral load of SARS CoV-2 with that of other respiratory viruses involved in coinfections. The mean viral load of SARS-CoV-2 was higher than that of the six co-detected respiratory viruses (*p* < 0.001). However, the mean viral load of the influenza virus was higher than that of SARS-CoV-2 and the six respiratory viruses involved in coinfections. These results indicate a more intensive replication of SARS CoV-2 and influenza viruses compared to that of other respiratory viruses involved in coinfections. The level of the viral load involved in mixed infections depends largely on the time of exposure to one virus relative to the other. The importance of this factor was minimized by estimating the average viral load of the individual viruses involved in coinfection. Our findings can be explained by positive or negative interactions between SARS-CoV-2 and other respiratory viruses *via* interferon-mediated or other immunological mechanisms. To our knowledge, such studies have not been conducted in other countries; therefore, we were unable to compare our results with those of other studies.

To evaluate the impact of coinfection on disease severity, we compared the frequency of clinical symptoms, laboratory parameters, treatment approaches, and disease outcomes in patients with SARS CoV-2 mono- and coinfection. The mean Ct value of SARS-CoV-2 was lower than that of other respiratory pathogens in cases of coinfections. Our findings contradict the opinion of some researchers who speculate that seasonal respiratory viruses (e.g., RV) have a shorter incubation period than SARS CoV-2 and induce the production of interferon, which inhibits SARS CoV-2 replication (38). Jeong et al. (24) reported that the median cycle threshold value of SARS-CoV-2 testing was slightly elevated in patients with coinfection. In another study, the differences in the mean Ct values between specimens with and without coinfection were not statistically significant (39). However, Burstein et al. (40) reported significant Ct differences (*p* < 0.01) between mono- and coinfected samples.

In the present study, fever, fatigue, and diarrhea were more frequently observed in patients with coinfections compared to those with monoinfections (*p* < 0.05). In coinfected febrile patients, the mean body temperature was higher than that in monoinfected patients (38.7° ± 0.7°C vs. 37.9° ± 0.6°C; *p*< 0.001). Patients with coinfections had longer hospital stays than those with a single SARS CoV-2 infection. The differences in CRP levels in the blood of mono- and coinfected patients >65 years of age were not statistically significant. No significant differences were observed in the treatment, need for high-flow oxygen therapy, or clinical outcomes. A similar comparison was made in a study in France, in which patients coinfected with SARS-CoV-2 and rhinovirus were significantly more likely to report a cough than those with SARS-CoV-2 monoinfection (62% vs. 31%; *p* = 0.0008) (10). However, a systematic review and meta-analysis reported no significant association between coinfection with SARS CoV-2 and influenza and any of the clinical symptoms, such as fever, cough, and dyspnea. With respect to laboratory parameters such as white blood cells, CRP, IL-6, and IL2R, no significant differences were observed between the coinfection and monoinfection groups. Only lymphocyte counts were significantly higher in the coinfection group (41). These contradictory results require further study to better understand the impact of coinfections with different respiratory viruses on clinical presentation and disease outcome.

This study has some limitations. First, the analysis includes data from only two hospitals, which may not represent the Bulgarian COVID-19 patient group as a whole. Second, it is also not possible to establish a sufficient relationship in terms of mortality in co-infected patients compared to such cases in monoinfected patients, as only one case of a co-infected patient with a fatal outcome was considered in this study. Third, we consider the role of coinfections with influenza viruses other than A(H3N2) to be omitted due to the lack of circulation of A(H1N1)pdm09 and influenza type B viruses during the study period of November 2020 to March 2022 in Bulgaria. We accept as an omission in our study the restrictions on the sample size, due to which deviations in the selection and the possibility of evaluation are possible.

In conclusion, we evaluated the rate and clinical impact of coinfection with SARS CoV-2 and other respiratory viruses in Bulgaria from November 2020 to March 2022. Our findings highlight the need for the maintenance of respiratory virus surveillance to understand the changing epidemiology of respiratory viruses during the period of relaxing public health and travel restrictions. The analysis of our clinical and laboratory data provided convincing evidence of the negative impact of coinfections on disease severity and outcome. In patients aged >65 years, coinfection with SARS CoV-2 and other respiratory viral pathogens, along with concomitant diseases, can worsen the clinical picture and lead to a fatal outcome. These studies should continue, as they are important for effective patient management and containment of the epidemic.

## Data availability statement

The original contributions presented in the study are included in the article/supplementary material, further inquiries can be directed to the corresponding author/s.

## Author contributions

IT conceptualized, designed the study. SA, RY, and TT were responsible for patient enrollment, assessment, and selection. IT, IM, SK, and NK performed the experiments. IT, IM, NK, SA, SV, RY, and TT analyzed the work results. IT and NK wrote the manuscript. IC reviewed the final manuscript. All authors read and approved the final manuscript.

## Funding

This article is supported by: contracts KΠ-06-H 43/5/30.11.2020/ Molecular-genetic and clinical characteristics of human coronavirus. Study of the role of SARS-CoV-2 in co-infections with other respiratory viruses, NO KΠ-0 6-DK1/7/29.03.2021/ Viral load, cytokines and serum antibody levels depending on the clinical severity of COVID-19 infection and by the European Regional Development Fund through Operational Program Science and Education for Smart Growth 2014–2020, Grant BG05M2OP001-1.002-0001-C04 Fundamental Translational and Clinical Investigations on Infections and Immunity.

## Conflict of interest

The authors declare that the research was conducted in the absence of any commercial or financial relationships that could be construed as a potential conflict of interest.

## Publisher's note

All claims expressed in this article are solely those of the authors and do not necessarily represent those of their affiliated organizations, or those of the publisher, the editors and the reviewers. Any product that may be evaluated in this article, or claim that may be made by its manufacturer, is not guaranteed or endorsed by the publisher.
